# Retrospective study on growth in infants with isolated Robin sequence treated with the Tuebingen Palate Plate

**DOI:** 10.1186/s13023-021-01959-2

**Published:** 2021-08-03

**Authors:** Cornelia Wiechers, Regina Iffländer, Rieke Gerdes, Melissa Ciuffolotti, Jörg Arand, Christina Weise, Katharina Peters, Bärbel Grandke, Siegmar Reinert, Bernd Koos, Christian F. Poets

**Affiliations:** 1 Department of Neonatology, Tuebingen University Hospital, Calwerstraße 7, 72076 Tuebingen, Germany; 2 Interdisciplinary Center for Craniofacial Malformations, Speech Therapy Centre, Tuebingen University Hospital, Hoppe-Seyler-Str. 3, 72076 Tuebingen, Germany; 3 Department of Orthodontics, Tuebingen University Hospital, Osianderstraße 2, 72076 Tuebingen, Germany; 4 Department of Craniofacial Surgery, Tuebingen University Hospital, Osianderstraße 2, 72076 Tuebingen, Germany; 5 Center for Cleft Palate and Craniofacial Malformations, Tuebingen University Hospital, Osianderstraße 2, 72076 Tuebingen, Germany

**Keywords:** Robin sequence, Pierre Robin, Infant, Growth, Nutrition, Feeding difficulties, Pre-epiglottic baton plate

## Abstract

**Background:**

Children with Robin sequence (RS) are at risk of growth failure, mainly due to their increased work of breathing and feeding difficulties. Various conservative and surgical treatment approaches exist, but their impact on weight gain has not yet been adequately addressed. A functional treatment concept, used in our center for > 20 years, includes a pre-epiglottic baton plate (Tuebingen palatal plate) and intensive feeding training.

**Objective:**

To investigate the effect of the Tuebingen treatment protocol on growth and weight trajectories during infancy.

**Methods:**

This retrospective study analyzed longitudinal data from infants with isolated RS admitted to Tuebingen University Children’s Hospital, Germany between 1998 and 2019. Through our electronic patient database, we evaluated anthropometric parameters until reaching 1-year follow-up. Results are shown as median (IQR).

**Results:**

In 307 infants analyzed, median Z-score for weight decreased from − 0.28 at birth to − 1.12 upon admission to our center at a median age of 22 days. Z-score then remained largely unchanged until discharge (Z-score difference, − 0.08), while the proportion of infants receiving tube feedings decreased from 55.1 to 13.7%. Z-score subsequently increased from − 1.17 at discharge to − 0.44 at the 1-year follow-up (*p* < 0.001).

**Conclusion:**

Based on a comparatively large cohort, this functional treatment was associated with better weight gain and improved feeding. As RS infants often show postnatal growth failure, weight monitoring may be a valuable parameter for monitoring treatment effectiveness.

*Clinical Trial Registration* Not necessary due to the retrospective design.

## Background

Robin sequence (RS), consisting of mandibular retrognathia, glossoptosis, upper airway obstruction (UAO) and optionally cleft palate [1, 2], has an estimated birth prevalence of 1:8500–1:14,000 [3, 4]. For several reasons, it also often leads to growth failure.

UAO results in an elevated energy expenditure due to an increased work of breathing, which is the main cause of growth failure in RS [1, 5], being further aggravated by feeding difficulties resulting from the characteristic anatomy. For example, glossoptosis may prevent placing the nipple on the body of the tongue during feeding, which together with mandibular micrognathia and an upper-lower jaw discrepancy may inhibit development of an efficient sucking pattern. In cleft palate, which is present in 80–90% of RS infants [3, 4], severity and size of the cleft usually correspond to the degree of feeding difficulties, because the wider the cleft palate, the smaller the area available for serving as an abutment for the nipple. It often also prevents achieving a sufficient oral vacuum for adequate sucking. As a result, the vast majority of mothers of babies with RS are unable to breastfeed [6, 7], and alternative feeding techniques often result in inadequate nutrient intake [2, 6, 8], so that reported rates of poor feeding in infants with RS range from 47 to 100% [5, 6, 8–12]. Further, infant feeding requires a complex coordination between breathing, sucking, and swallowing, which can be disrupted by neurologic problems also seen in some infants with RS [7, 13]. Besides these functional impairments, growth retardation may also be related to an underlying syndrome, chromosomal or other abnormalities that occur in up to 50% or RS infants [1, 11, 14].

Impaired weight gain in the first postnatal months has been reported in several studies on RS [5, 11, 15–17]. Malnutrition is defined as an imbalance between nutrient requirements and intake that results in cumulative deficiencies in energy, protein or micronutrients that can negatively impact growth, development and other relevant outcomes [18]. Therefore, identification and subsequent treatment of upper airway obstruction and feeding difficulties is particularly important in RS, especially since failure to thrive is associated with impaired neurodevelopment, at least in preterm infants [19]. Treatment protocols vary widely in RS, ranging from non-surgical (e.g., prone positioning, insertion of a nasopharyngeal tube or continuous positive airway pressure) to operative procedures (e.g., tongue-lip adhesion, mandibular distraction osteogenesis or tracheostomy) [1, 20]. However, their impact on weight gain has not yet been adequately studied or compared.

Our group has developed and implemented a treatment protocol consisting of an individualized orthodontic appliance (Tuebingen Palate Plate (TPP)), feeding training and orofacial stimulation therapy, which addresses the two main clinical problems in RS, i.e. UAO [21–23] and feeding difficulties [5]. The aim of this audit was to determine growth and weight trajectories in the first year of life in a large cohort of infants with isolated RS treated according to this protocol.

## Methods

### Participants

This is a retrospective single center study of infants with isolated RS admitted to Tuebingen University Children’s Hospital between 1/1998 and 12/2019. Our hospital is a national referral center for RS and other craniofacial malformations, to which most patients are referred by other hospitals after their attempted therapy (e.g., prone positioning) has failed. For this analysis, infants with an underlying syndrome (including Stickler syndrome or chromosomal defects such as trisomy 18 or 21) or other severe malformations were excluded. Clinical data were collected from the department’s electronic database, medical records and an ongoing inventory of RS patients kept by one of the authors (RI). Anthropometric measurements were analyzed at five time points: at birth, upon admission to our center, prior to discharge, at the first follow-up after this initial discharge and at a 1-year follow-up. Regarding treatment epochs, infants were divided into 3 groups by their date of first admission: 1998–2005, 2006–2012 and 2013–2019.

Body mass was measured to the nearest 0.1 g using a digital scale (Soehnle, Backnang, Germany), length to the nearest 0.1 cm using a recumbent, digital infant length board (Ulmer Stadiometer, Busse, Ulm, Germany) and head circumference to the nearest 1 mm using a non-stretchable tape measure. Calculation of Z-scores for weight, length and head circumference was based on normal values for age as reported by the World Health Organisation (WHO) [24]; these parameters were computed using Perccalc® (Paedsoft, Tuebingen, Germany).

### Tuebingen treatment protocol

Our treatment protocol, based on an interdisciplinary team of neonatologists, pediatric sleep specialists, neonatal nurses, speech therapists, orthodontists and craniomaxillofacial surgeons, has been described in detail before [20, 21, 23, 25].

In brief, following admission, the severity of UAO is assessed by a multichannel baseline cardiorespiratory sleep study (polygraphy). Indication for initiating TPP treatment is a mixed-obstructive apnea index (MOAI) > 3 in this initial sleep study [21, 23, 26]. An individualized orthodontic palatal plate with a velar extension, the TPP, is produced with the tip of this velar extension ending just above the epiglottis, as confirmed by awake fiberoptic nasopharyngoscopy. The TPP is supplemented by early specialized oral feeding techniques, starting with an infant feeder (Finger Feeder, Medela, Baar, Switzerland) and followed by a special feeding bottle with variable milk flow (Playtex Drop-Ins®, Playtex Products, Edgewell, North Bergen, NY) and stimulation of the oral musculature based on the Castillo-Morales® approach [27, 28]. The TPP is continuously worn, with its fit being regularly checked by the nursing team, and removed and cleaned at least once daily. After a few days of treatment with a clinically well-fitting TPP, its effectiveness in relieving UAO is confirmed by a second sleep study, with the aim of achieving a MOAI < 3. If this sleep study still shows a MOAI > 3, the angle between the base of the plate and its velar extension is modified. Parents are involved from the beginning in handling the TPP and in feeding techniques. Adequate oral intake allowing removal of the nasogastric tube and weight gain are monitored for a few days before infants are discharged home.

After discharge, RS infants are re-admitted for clinical, orthodontic and sleep-study follow-up in three-monthly intervals throughout their first year of life. Due to cranial physical growth, infants usually require a larger TPP 3 months after discharge. For this second and usually last TPP fitting, an inpatient stay of approximately 3 days is usually necessary, followed by another polygraphy. At 6–8 months of age, TPP treatment is usually discontinued if the facial profile has harmonized and sleep study results show a MOAI < 1. Surgical closure of the cleft palate is done at around 12 months of age.

### Sleep studies

Cardiorespiratory sleep studies are performed using a computerized polysomnographic system (Embla N 7000, MedCare, Reykjavik, Iceland); the study montage and evaluation criteria have been described elsewhere [9, 14, 21]. Sleep studies are always performed in the supine position before and after the TPP has been applied. Since 2003, our sleep study evaluation has been standardized according to the criteria of the American Academy of Sleep Medicine (AASM) [29, 30], sleep study results were therefore only included for evaluation from 2003 onwards. Central, mixed and obstructive apneas are identified and a mixed-obstructive apnea index (MOAI) calculated as the sum of all mixed and obstructive apneas per hour of total sleep time.

### Ethics

This study was approved by the ethics committee of Tuebingen University Hospital, which included a waiver of parental consent (reference number 435/2019 BO2).

### Statistical analysis

Data are presented as mean (standard deviation (SD)) if normally distributed, or as median and interquartile range (IQR) if not. The Shapiro–Wilk test was used to test for a possible normal distribution of the data. In case that within a table a minority of parameters were normally distributed, all data in that table are presented as median (IQR) to improve clarity of presentation. The Kruskal–Wallis test was administered to test the hypothesis that all groups come from the same distribution if there were more than two independent, non-normally distributed samples. Alternatively, if there were exactly two independent, non-normally distributed samples, the Mann–Whitney test was used. A significance level of 5% was used, so that a significant difference between the groups was assumed at *p* < 0.05. The Spearman rank correlation was used to determine the relationship between quantitative variables. Comparisons of Z-score values for weight between various time points were performed using the t-test. To account for the impact of intrauterine growth restriction, Z-score differences for weight (e.g., Z-score_discharge_ − Z-score_admission_) were calculated to illustrate weight gain. Analyses were performed with IMB SPSS Statistics Version 25, Microsoft Excel, GraphPad Prism® 8.1.0 (GraphPad Software, San Diego, CA, USA).

## Results

### Participants

Between 1998 and 2019, a total of 307 infants (53% girls) with isolated RS were admitted to our center. Only a minority of 29 (9.6%) was inborn; median age at admission to our center was 22 days and median duration of the initial hospital stay 18 days.

Of these 307 infants, 4 (1.3%) did not require treatment due to a MOAI < 3 in the initial sleep study and only a very mild degree of glossoptosis or mandibular retrognathia; in 3 (1%) infants, TPP therapy was discontinued due to side effects (recurrent pressure marks). In the remaining 300 (97.7%) patients, UAO was successfully treated, i.e. the MOAI, available in 246 infants, decreased from 9.0 (3.4–22.8) at admission to 0.9 (0.3–1.9) at discharge (median (IQR); *p* < 0.001). No infant required craniofacial surgery or tracheostomy; 2 children, referred with a pre-existing tracheostoma, had this subsequently closed. Demographics are shown in Table 1.

**Table 1:** Baseline characteristics of infants with isolated RS

Variable	N patients with data	n (%)/median (IQR)
Female	307	161 (53%)
Cleft palate	307	260 (86%)
Inborn	307	29 (9.6%)
Gestational age at birth (weeks)	292	39.1 (37.9–40.1)
Birth weight (g)	296	3200 (2759–3560)
Small for gestational age	296	35 (11.6%)
Birth head circumference (cm)	277	34.5 (33.0–35.5)
5 min APGAR score	205	9 (8–10)
Age upon admission^a^ (days)	307	22 (5–55)
Age at discharge^a^ (days)	307	44 (24–80)
Duration of hospital stay^a^ (days)	307	18 (13–28)

*CPAP* continuous positive airway pressure, *SGA* small for gestational age defined by a Z-Score < −1.282

^a^Initial hospitalization in our center with first fitting of a Tuebingen palatal plate

### Growth

A total of 1326 weight measurements were collected in 307 infants at the different time points. At birth, the median Z-score weight was − 0.28, with 35 (11.6%) infants being small for gestational age (Z-score < −1.28). Pronounced postnatal growth failure occurred until the first admission to our center (Z-score difference of − 0.77) (Table 2). The Z-score difference for weight between admission and discharge was − 0.08. Also, after discharge, there was no further growth failure until the first follow-up at a median age of 120 days (Z-score difference − 0.05); to the contrary, Z-scores improved from − 1.17 at discharge to − 0.44 at the 1-year follow-up (*p* < 0.001).

**Table 2:** Weight parameters until 1-year follow-up

	N (%)	Age (days)	Weight (kg)	Z-score weight	Difference in Z-score for weight			
Birth	296 (96.4%)	–	3.20 (2.76–3.56)	− 0.28 (− 0.86 to 0.33)				
Admission	307 (100%)	22 (5–55)	3.46 (3.05–4.03)	− 1.11 (− 1.80 to − 0.35)	Admission—Birth	− 0.77 (− 1.24 to − 0.36)		
Discharge	307 (100%)	44 (24–80)	4.00 (3.57–4.68)	− 1.17 (− 1.76 to − 0.50)	Discharge—Birth	− 0.90 (− 1.28 to − 0.55)	Discharge-Admission	− 0.08 (− 0.34 to 0.18)
First follow-up	240 (78.2%)	120 (84–163)	5.63 (4.81–6.42)	− 1.09 (− 1.66 to − 0.43)	First follow-up—Birth	− 0.81 (− 1.47 to − 0.28)	First follow-up—Admission	− 0.05 (− 0.61 to 0.64)
One-year follow-up	183 (59.6%)	326 (292–373)	8.50 (7.59–9.50)	− 0.44 (− 1.13 to 0.15)	One-year follow-up—Birth	− 0.25 (− 0.97 to 0.44)	One-year follow-up—Admission	0.60 (− 0.27 to 1.34)

Values are given as median (IQR) or n (%)

Upon admission to our center, infants with severe obstructive sleep apnea syndrome (OSAS; MOAI > 10) showed more severe postnatal growth failure (Z-score difference − 0.86 vs − 0.68, *p* = 0.0025) and a lower Z-score difference between birth and first follow-up (− 0.75 vs − 1.0, *p* = 0.046). OSAS severity, i.e. the MOAI before (and after) onset of TPP treatment, had no effect on further weight gain. Patients hospitalized at our center at < 4 weeks of age showed less severe growth failure than those admitted at 4–8 weeks or > 8 weeks of age (− 0.63, − 1.04 vs. − 1.43, *p* < 0.0001, respectively). However, Z-score differences at the first or the 1-year follow-up were not influenced by age at first TPP fitting. In the last treatment period (2015–2019), infants showed a significantly better weight gain between birth and the 1-year follow-up than in the first period (1998–2005; Z-score difference for weight − 0.05 vs − 0.58, respectively).

Postnatal growth failure was significantly more common in boys upon admission to our center compared to girls (Z-score difference for weight − 0.87 vs − 0.73, p = 0.009). Otherwise, no sex changes were found for weight or Z-score differences between birth and the first or the 1-year follow-up.

### Nutrition and feeding

The proportion of infants requiring nasogastric tube feeding decreased significantly from 55.1% at the initial hospital admission to 13.7% at discharge (Table 3). Upon first discharge home at a median age of 44 days, 23.8% of infants were fed exclusively with mother's own milk, and 25.1% received a combination of mother's own milk and formula. Feeding supplements, e.g. Ceres oil, Maltodextrin® (Nutricia, Erlangen, Germany) or Duocal® (Nutricia, Erlangen, Germany), were used in 19.5% of infants at discharge.

**Table 3:** Infant feeding during initial hospitalization

Feeding difficulties (NGT)	Upon admission n = 306	At discharge n = 307
No tube	137 (44.6%)	265 (86.3%)
Nasogastric tube	166 (54.1%)	36 (11.7%)
Nasoduodenal tube	0 (0%)	1 (0.3%)
PEG	3 (1.0%)	5 (1.6%)

*NGT* nasogastric tube, *PEG* percutaneous endoscopic gastrostomy

## Discussion

In this study, we analyzed growth data in a cohort of infants with isolated RS treated with the TPP. To our knowledge, this is the largest study reporting growth and weight gain for infants with isolated RS.

Infants showed considerable growth failure between birth and admission to our center (Z-score difference of − 0.77), confirming a high risk of growth failure in infants with isolated RS. TPP’s immediate effect of correcting UAO may explain the reversal of this trend following admission to our center [9, 22]. Infants even showed some catch-up growth after hospital discharge, indicated by a decrease in their median Z-score difference for weight to − 0.25 at the 1-year follow-up. This improvement in weight gain was particularly pronounced in the most recent analysis period (2013–2019), probably due to us paying more attention to this aspect.

Infants with RS are generally at increased risk of growth failure [1, 5, 11]. Various conservative and surgical treatment approaches exist, but there is limited data on their impact on weight gain. A study involving 74 infants with RS, mostly treated conservatively with prone/lateral positioning, reported a body weight < P10 in one quarter of cases at 6–24 months of age [31]. Malnutrition with a Z-score for weight of < −1 at discharge was also present in 57% of RS patients in an Australian study with 49 RS patients mainly treated conservatively. Here, Z-score decreased from 0.21 to − 1.27 between birth and discharge [16]. In a prospective, population-based survey on interventions used in RS infants in Germany, non-surgical treatment was also the preferred choice, with TPP-treated infants showing significantly better weight gain during their first hospital stay than those receiving other treatments (SDS difference for weight − 0.37 vs − 0.74) [5].

A slower growth rate compared to WHO norms was also observed in 24 RS infants predominantly treated with mandibular distraction osteogenesis. These infants showed a marked decrease in growth rate in the first 6 months after birth, with Z-scores at 3 and 5 months of age being − 2.19 and − 1.64, respectively [17]. After a subsequent period of accelerated growth in most infants, boys had caught up with their unaffected peers by 12 months of age, while girls continued to show a weight below WHO norms [17]. In a Dutch study on 69 infants with RS, growth in the first 2 years was significantly lower than in patients with isolated cleft palate [11]. Within the RS cohort, neither the presence of associated disorders (isolated/non-isolated) nor the type of intervention (surgical/conservative) had a significant effect on growth [11].

Unfortunately, no sleep study results were reported in most of the above studies, so it is unclear how effectively UAO had been treated. In addition to sleep studies, laryngoscopy or magnetic resonance imaging (MRI) may be useful to visualize the severity and cause of UAO (e.g. glossoptosis) [32, 33]. In our cohort, infants with effectively treated OSAS, despite a severely abnormal initial sleep study result (MOAI > 10), were growing equally well as less severely affected infants. Our study illustrates that with appropriate treatment of UAO and feeding difficulties thanks to our functional treatment approach including the TPP, further growth failure is prevented, and catch-up growth becomes possible during the first year of life. Overall, adequate weight gain in RS infants is of particular interest, especially as growth restriction in the first postnatal months was shown to negatively impact on neurocognitive development, at least in preterm infants [19].

In our cohort, the proportion of infants requiring nasogastric tube feeding was reduced from 55% at admission to 14% after a median hospital stay of 18 days. Nevertheless, Z-score for weight remained unchanged between admission and discharge (Z-score difference − 0.08) and also until the first follow-up at 4 months of age (Z-score differences − 0.05). We speculate that these encouraging results may be explained by the functional orthodontic effect of the TPP helping the tongue to assume its physiologic position, thereby facilitating oral feeding. Furthermore, combining independent oral feeding with functional exercises is considered an important factor in strengthening the orofacial musculature and possibly supporting mandibular catch-up growth as suggested by the “form follows function” paradigm [34]. In 2017, a systematic review reported feeding difficulties in 80% (range 47–100%) of RS infants, and long-term nasogastric tube feeding in 55% (range 11–100%) [11]. Mean duration of tube feeding ranged from a few weeks [9, 11, 12] to several months [7, 35, 36].

In our study, 24% of infants were exclusively fed their mothers’ own milk at discharge, and 25% received a combination of mothers’ own milk and formula. This proportion of exclusively breastmilk-fed infants with isolated RS is significantly lower than that in the general population [37–39] or as recommended by the WHO [40]. In the above Australian study, the rate of exclusive breast milk feeding in the first two postnatal months was 21% in conservatively treated infants and 9% in those receiving jaw distraction surgery [16]. Time-consuming milk pumping, a low likelihood of exclusive breastfeeding, stressful circumstances with a hospitalized infant, and increased need for care were cited as underlying reasons.

Our study has some limitations. First, it is retrospective and there is a significant proportion of missing follow-up data, especially during the first analysis period, when our treatment concept was still being developed. Some loss of post-discharge follow-up examination results can also be explained by a high proportion of families living far away from our center. Additional information, e.g. the prenatal detection rate in routine ultrasound screening during pregnancy, incidence of feeding difficulties after discharge and compliance with using the TPP, would have been helpful in retrospect to identify other important factors related to growth. Also, some data from sub-samples have already been reported elsewhere [9, 20, 21, 34], but all of these earlier reports covered only the first 3 months after discharge, none had its focus on weight gain, and the period covered as well as the number of patients included were less than half of what has been included in the present manuscript.

Strengths of this study include its large sample size despite the rarity of isolated RS and consistency over time in our unified treatment approach. Ideally, a standardized neurocognitive examination of all children with RS at the age of 2 and 6 years would be appropriate, not least to assess the influence of growth failure on cognitive function.

## Conclusions

This retrospective study in a cohort of infants with isolated RS indicates that catch-up after initial growth failure is possible in RS infants after implementing effective treatment of UAO and feeding training. Insufficient weight gain may be an early sign of residual UAO and/or a feeding disorder and could adversely affect development. Therefore, growth failure in RS infants should be anticipated, followed and treated adequately and promptly.

## Data Availability

De-identified individual data will not be made available, because trial subjects have not been asked to consent.

## Abbreviations

MOAI: Mixed-obstructive apnea index
OSAS: Obstructive sleep apnoea syndrome
RS: Robin sequence
TPP: Tuebingen Palatal Plate
UAO: Upper airway obstruction
